# Effects of ultraviolet B exposure on DNA methylation in patients with systemic lupus erythematosus

**DOI:** 10.3892/etm.2013.960

**Published:** 2013-02-18

**Authors:** XIAOHUA ZHU, FENG LI, BO YANG, JUN LIANG, HAIHONG QIN, JINHUA XU

**Affiliations:** Department of Dermatology, Huashan Hospital, Fudan University, Shanghai 200040, P.R. China

**Keywords:** lupus erythematosus, systemic, DNA methylation, DNA methyltransferase 1, methyl CpG binding domain protein 2

## Abstract

The aim of this study was to investigate the effects of ultraviolet B (UVB) exposure on DNA methylation in patients with systemic lupus erythematosus (SLE) and its significance in the pathogenesis of SLE. T cells from 35 SLE patients and 21 healthy individuals were cultured and irradiated with UVB. The global DNA methylation profiles of the T cells obtained from the patients and controls following irradiation with UVB were assessed using specific monoclonal antibodies for 5-methylcytosine and analyzed quantitatively through flow cytometry. Real-time reverse transcription-polymerase chain reaction (RT-PCR) was used to analyze the levels of DNA methyltransferase 1 (DNMT1) and methyl CpG binding domain protein 2 (MBD2) in T cells from the patients and controls following UVB irradiation. Significant global DNA hypomethylation was observed in the SLE patients compared with the controls (P<0.01). The SLE patients also had significantly lower levels of DNMT1 mRNA expression (P<0.01) and significantly higher levels of MBD2 mRNA compared with the controls (P<0.01). DNA methylation was decreased following UVB irradiation at two different dosages and the DNA methylation levels of the patients with active SLE were more sensitive to UVB. The level of DNMT1 mRNA was decreased following UVB irradiation at the higher dosage in the patients with active SLE, but no significant difference was observed in MBD2 mRNA expression. UVB exposure is able to inhibit DNA methylation and DNMT1 mRNA expression, which is subsequently involved in the epigenetic mechanism of SLE. The process by which DNA hypomethylation occurs in patients with SLE is complicated and the multiple factors that are involved in DNA methylation and demethylation events require further study.

## Introduction

Systemic lupus erythematosus (SLE) is a chronic relapsing autoimmune disease characterized by the production of auto-antibodies directed against a range of cellular components and its pathogenesis remains unclear. Genetic, hormonal, environmental and immunological factors are involved in the pathogenesis of SLE (1,2).

It is known that epigenetic modifications, including DNA methylation and histone modifications, have a significant impact on gene expression and abnormal decreases in T-cell DNA methylation have been implicated in the development of drug-induced and idiopathic lupus (3,4). The decreased T-cell DNA methylation results in the overexpression of methylation-sensitive genes, such as CD11a (LFA-1), perforin and CD70, in the T cells and this leads to T-cell autoreactivity *in vitro* and autoimmunity *in vivo* (5–9).

The methylation status of DNA is associated with three types of enzymes, which have effects on maintenance methylation, *de novo* methylation and demethylation (10,11). DNA cytosine-5-methyl transferase 1 (DNMT1) is involved in maintaining methylation, while methyl CpG binding domain protein 2 (MBD2) is associated with demethylation (12). The methylation-related molecules, DNMT1 and MBD2, may also be associated with the development of SLE.

Environmental effects on lupus may have a role in mediating epigenetic changes in immunity. Exposure to ultraviolet (UV) light has long been associated with the exacerbation of SLE and photosensitivity remains a diagnostic criterion for this disease (13), with up to 73% of patients with SLE reporting photosensitivity (14). Most, but not all, cutaneous lupus lesions occur in light-exposed areas and may be triggered by sunlight exposure. Sunlight exposure itself is able to induce systemic disease activity (15).

UV irradiation, particularly ultraviolet B light (UVB, 290–320 nm), may induce systemic disease activity. However, it remains unclear whether UVB affects SLE by altering DNA methylation. Thus, the elucidation of the mechanisms of the effects of UVB on DNA hypomethylation in SLE patients may provide clues to the pathogenesis of the SLE. To investigate these mechanisms, we analyzed the DNA methylation status and gene expression of DNMT1 and MBD2 in T cells from SLE patients and healthy controls following treatment with different dosages of UVB irradiation. The present study reports the findings in this field.

## Materials and methods

### Human subjects

Patients with SLE (n=35) were recruited from the outpatient and inpatient services at the Huashan Hospital, Fudan University (Shanghai, China). They included 31 females and 4 males (mean age: 33.4 years; range, 18–51 years). A total of 21 gender- and age-matched healthy volunteers served as the controls (18 females and 3 males; mean age 32.7 years; range, 19–51 years). This study was approved by the institutional review board of the Huashan Hospital (IRB: KY2009-054) and written informed consent was obtained from all participants. All patients with SLE fulfilled at least four of the criteria of the American Rheumatism Association for the classification of SLE (13) and disease activity was assessed using the SLE Disease Activity Index (SLEDAI) (16). Active disease was defined as an SLEDAI score ≥5. In the 35 patients, 16 had the active disease, while the other 19 were stable.

### Peripheral blood mononuclear cells and T-cell isolation

Peripheral blood mononuclear cells were isolated using density gradient centrifugation. T cells were isolated by CD3^+^ magnetic beads according to the manufacturer’s instructions (T cell isolation kit; Miltenyi Biotec, Bergisch Gladbach, Germany).

### T-cell culture and UVB irradiation

CD3^+^ T cells were cultured in RPMI-1640 medium supplemented with 10% heat-inactivated fetal bovine serum (FBS), 2 mM sodium pyruvate, 100 IU/ml penicillin and 100 *μ*g/ml streptomycin.

UVB irradiation was performed using Waldman UV236B lights (Waldmann Lighting Ltd., Wheeling, IL, USA), which emit the majority of their energy within the UVB range (290–320 nm) with an emission peak at 311 nm. CD3^+^ T cells were irradiated in PBS with different doses of UVB (0, 50 and 100 mJ/cm^2^). PBS was removed following irradiation and RPMI-1640 containing 10% FBS was then added and the cells were cultured for 24 h. The doses of UVB were selected based on the World Health Organisation guidelines for sun exposure (17) and on the standard erythemal dose (SED), a cumulative measure of erythemal or sunburning solar UV irradiation (18). Following culturing, CD3^+^ T cells were harvested for the analyses of DNA methylation and mRNA levels.

### Flow cytometric analysis of 5-methylcytosine staining

Global DNA methylation was evaluated by staining the cells with a specific monoclonal antibody against 5-methylcytidine using previously published methods (19,20). The T cells were washed with phosphate-buffered saline (PBS) supplemented with 0.1% Tween-20 and 1% bovine serum albumin (PBST-BSA), fixed with 0.25% paraformaldehyde at 37°C for 10 min and 88% methanol at −20°C for at least 30 min. After two washes with PBST-BSA, the cells were treated with 2 M HCl at 37°C for 30 min and then neutralized with 0.1 M sodium borate (pH 8.5). The cells were blocked with 10% goat serum in PBST-BSA for 20 min at 37°C and incubated with anti-5-methylcytidine antibody (1 *μ*g/ml) for 45 min at 37°C, followed by staining with goat anti-mouse IgG conjugated with fluorescein isothiocyanate (Santa Cruz Biotechnology, Inc., Santa Cruz, CA, USA). Finally, the cells were washed three times with PBS and resuspended in PBS for analysis by flow cytometry.

### DNMT1 and MBD2 real-time reverse transcription-polymerase chain reaction (RT-PCR)

Real-time quantitative RT-PCR was performed using an iCycler IQ5 (Bio-Rad, Hercules, CA, USA) and previously published methods (21). Total cellular RNA was isolated from T cells using the TRIzol reagent (Invitrogen Life Technologies, Carlsbad, CA, USA). The RNA extracts were resuspended in 15 *μ*l RNase-free water and the RNA concentration was adjusted to ∼1 *μ*g/*μ*l and used for cDNA synthesis. The final reaction volume was 25 *μ*l consisting of 12.5 *μ*l Maxima SYBR-Green master mix (Fermentas, Thermo Scientific, Waltham, MA, USA), 0.5 *μ*l each specific primer (10 *μ*M), 1 *μ*l cDNA template and 10.5 *μ*l nuclease-free H_2_O. The cycling conditions were 5 min at 94°C; then 40 cycles of 30 sec at 94°C, 30 sec at 55°C and 30 sec at 72°C. The DNMT1 and MBD2 mRNA levels were quantified relative to β-actin transcripts. The following primers were used: DNMT1 forward: 5′-GATTTGTCCTTGGAG AACGGTG-3′ and reverse: 5′-TGAGATGTGATGGTGGTT TGCC-3′; MBD2 forward: 5′-AGGTAGCAATGATGA GACCCTTTTA-3′ and reverse: 5′-TAAGCCAAACAGCAG GGTTCTT-3′; β-actin forward: 5′-GCACCACACCTT CTACAATGAGC-3′ and reverse: 5′-GGATAGCACAGCCTG GATAGC AAC-3′.

### Statistical analysis

The data were analyzed using the Student’s t-test, regression analysis or analysis of variance (ANOVA) as appropriate, calculated with Stata 7.0 software. P<0.05 was condsidered to indicate a statistically significant result.

## Results

### Patterns of DNA hypomethylation in SLE patients

**Figure 1:**
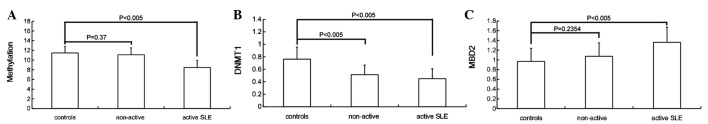
Comparison of (A) DNA methylation and mRNA levels of (B) DNMT1 and (C) MBD2 between healthy controls, patients with active SLE and patients with non-active SLE. SLE, systemic lupus erythematosus; DNMT1, DNA methyltransferase 1; MBD2, methyl CpG binding domain protein 2.

To determine whether global DNA methylation was decreased in SLE patients, T cells were isolated from 35 patients and 21 normal controls. DNA hypomethylation was observed in the patients with SLE, whose DNA methylation level was 9.892±1.939 compared with 11.479±1.291 for the controls, and the difference was statistically significant (P= 0.0016). Statistically significant differences in DNA methylation were observed between the active (SLEDAI ≥5) and non-active patients (SLEDAI <5; 8.469±1.458 vs. 11.090±1.418, respectively; P<0.005), while no apparent differences were observed between the patients with non-active SLE and controls (P=0.37; Fig. 1). There were no statistically significant differences between males and females in the patients or in the control group. In the present study, no correlation was observed between the methylation value and the patient’s age, although other studies (22) have reported age-related changes in DNA methylation.

### Comparison of DNA methylation following various dosages of UVB radiation

The DNA methylation levels following irradiation with various dosages of UVB are shown in Table I. The level of DNA methylation decreased following UVB irradiation in the SLE patients and controls. The reduction in DNA methylation was statistically significant following irradiation with 50 mJ/cm^2^ UVB in the patients with active SLE (P<0.005), while the decrease was statistically significant following irradiation with 100 mJ/cm^2^ UVB in the non-active SLE patients (P<0.005) and controls (P<0.005). It was observed that the patients with active SLE had markedly lower levels of DNA methylation (P<0.005) following irradiation with 100 mJ/cm^2^ UVB.

### mRNA levels of DNMT1 and MBD2 in SLE patients

Quantitative real-time PCR assays were performed to evaluate the mRNA levels of DNMT1 and MBD2 in the T cells. β-actin was selected as a control for normalizing the mRNA levels, as its expression does not change in the T cells of patients with lupus (23). Patients with SLE had significantly lower levels of DNMT1 mRNA (0.484±0.158) compared with the controls (0.762±0.194) and the difference was statistically significant (P<0.005). The levels of DNMT1 mRNA were lower in active patients (SLEDAI ≥5), but there was no significant difference between the active and non-active patients (SLEDAI <5; 0.449±0.160 vs. 0.514±0.154, respectively; P=0.2352). The MBD2 mRNA levels were significantly higher in the patients with active SLE compared with the non-active SLE patients (1.360±0.310 vs. 1.074±0.274, respectively; P=0.0066), while there was no significant difference between the controls (0.970±0.269) and non-active patients (P=0.2354; Fig. 1). There was no correlation between the levels of mRNA and the age of the patient.

### Comparison of mRNA levels of DNMT1 and MBD2 following various dosages of radiation

The mRNA levels of DNMT1 and MBD2 following various dosages of UVB irradiation are shown in Tables II and III, respectively. The expression of DNMT1 decreased following 100 mJ/cm^2^ UVB irradiation in the patients with active SLE and the difference was statistically significant (P=0.0166). No significant differences were observed in the expression of DNMT1 following various dosages of UVB irradiation (50 mJ/cm^2^ and 100 mJ/cm^2^) in the non-active SLE patients (P>0.05) and the controls (P>0.05). There were no significant difference in the levels of MBD2 mRNA following various dosages of UVB irradiation in the patients (active SLE and non-active SLE) and controls (P>0.05).

### Correlations between DNA hypomethylation, levels of DNMT1 and MBD2 mRNA and disease activity

**Figure 2:**
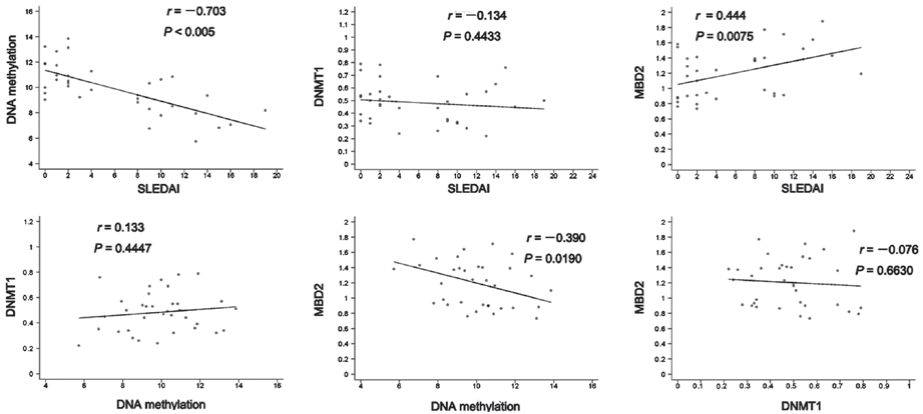
Correlations between DNA methylation, mRNA levels of DNMT1 and MBD2 and SLEDAI in patients with systemic lupus erythematosus. DNMT1, DNA methyltransferase 1; MBD2, methyl CpG binding domain protein 2: SLEDAI, systemic lupus erythematosus disease activity index.

The associations between the DNA hypomethylation and the mRNA levels of DNMT1 and MBD2 in the SLE patients were analyzed. DNA methylation showed an inverse correlation with SLEDAI in the SLE patients and the correlation was statistically significant (r=−0.703, P<0.005). However, no positive or negative correlations with DNA methylation were observed for the DNMT1 levels in the SLE patients. A negative correlation was observed between MBD2 expression and DNA methylation in the patients with SLE (r=−0.39, P=0.019). Furthermore, the level of MBD2 expression was positively correlated with SLEDAI (r=−0.444, P=0.0075). No correlation between the mRNA levels of DNMT1 and MBD2 was observed in the SLE patients (r=−0.076, P=0.663; Fig. 2).

In addition, whether medications (such as prednisone and cyclophosphamide) are responsible for T-cell DNA hypomethylation and the changes in the mRNA levels of DNMT1 and MBD2 was investigated. No statistically significant differences were observed between the treated and untreated groups with respect to either DNA methylation or the mRNA levels of DNMT1 and MBD2.

Following UVB irradiation, the DNA methylation level in the patients with active SLE (SLEDAI ≥5) was significantly lower compared with that of the non-active SLE patients (SLEDAI <5). The DNA methylation levels of all patients decreased following UVB irradiation irrespective of whether the patients were treated. Furthermore, the mRNA levels of DNMT1 decreased in the patients with active SLE following UVB irradiation, irrespective the treatment with medications such as prednisone and cyclophosphamide.

Whether DNA methylation was correlated with laboratory parameters, such as anti-dsDNA titers, and C3 and C4 concentrations, was also studied. No statistically significant positive or negative correlations were observed between DNA methylation and the laboratory parameters. Moreover, no differences were observed between these groups with regard to the mRNA levels of DNMT1 and MBD2.

## Discussion

SLE is a chronic relapsing autoimmune disorder characterized by multiple T-cell biochemical abnormalities and autoantibody production (24). However, the mechanisms underlying idiopathic SLE remain unclear. Previous studies have indicated that genetic and environmental factors contribute to the pathogenesis of SLE.

DNA methylation refers to the addition of a methyl group to position 5 of the cytosine ring. This process, catalyzed by a group of DNA methyltransferase, occurs in CG pairs, usually clustered within or around promoter sequences to form CpG islands. DNA methylation is an epigenetic process linked to the regulation of several biological events, including embryonic development, transcriptional regulation of gene expression, X chromosome inactivation, genomic ‘imprinting’, chromatin modification and silencing endogenous retroviruses (25,26). Any changes in the pattern of DNA methylation in the promoters of mature cells may have pathological consequences. Changes to the methylation pattern have been reported to contribute to the development of several diseases (10,11) and certain hereditary immunodeficiency disorders are known to be associated with abnormal methylation patterns (27,28). It has been demonstrated that the adoptive transfer of T cells made auto-reactive by treatment with DNA methylation inhibitors (29,30) is sufficient to cause a lupus-like disease in unirradiated syngeneic mice. A growing body of evidence suggests a role for abnormal T-cell DNA methylation in causing drug-induced and idiopathic lupus (31).

The underlying mechanism by which hypomethylated DNA exists in SLE patients remains unclear. DNMT1 maintains the levels and patterns of methylated DNA during mitosis, which has a five- to 30-fold preference for hemimethylated substrates (32), while DNA methyltransferase (DNMT) 3A and 3B (DNMT3B) are responsible for *de novo* DNA methylation during development (33). Certain studies (32,34) have observed that T cells from patients with active lupus have diminished DNMT1 mRNA levels. The observation that inhibiting ras-MAPK signaling decreases DNMT1 expression to the same extent as is observed in lupus and that this decrease correlates with DNA hypomethylation *in vitro* and *in vivo*, suggests that the defect in DNMT1 is the primary reason for DNA hypomethylation in SLE (35).

Several types of methyl-CpG binding proteins (MBDs) have been identified, including MBD1, MBD2, MBD3, MBD4, MeCp2 and Kaiso (36,37). MBD2 binds methylated cytosine and attracts chromatin inactivation complexes containing histone deacetylase. MBD2 is able to demethylate DNA (12,38) and is also a transcriptional repressor of genes (39).

UV irradiation has been shown to lead to specific demethylation events during subsequent rounds of replication (40). As a consequence of aberrant repair or repair methylation, UV irradiation has also been used to activate the transcription of a quiescent metallothionine gene, which, based on 5-azacytidine-reactivation experiments, is considered to be under methylation control (41). In clinical practice, UV irradiation, particularly with UVB light (290–320 nm), may induce a flare-up of lupus disease activity (42).

To investigate the role of UVB in the pathogenesis of SLE, the DNA methylation levels and mRNA levels of DNMT1 and MBD2 were first analyzed in patients with SLE and in controls without UVB irradiation. The results showed that, without UVB irradiation, the patients with SLE had significantly lower DNA methylation levels, lower expression levels of DNMT1 and higher expression levels of MBD2 mRNA compared with the controls. Certain studies (42) have observed that the levels of DNMT1 decrease with aging and that this decrease is correlated with the hypomethylation of sequences flanking the ITGAL promoter, a gene which encodes CD11a, a subunit of LFA-1. This hypomethylation may be a contributing mechanism to increased T-cell gene expression and the development of autoimmunity with aging (29). In the present study, no correlation existed between DNMT1 mRNA levels and SLEDAI and no correlation was observed between DNMT1 mRNA levels and age in the control group and SLE patients without UVB irradiation, while the levels of DNMT1 mRNA were similar in males and females. In the present study, an inverse correlation was observed between methylation and SLEDAI in the patients without UVB irradiation, while no correlation was observed between methylation and DNMT1 mRNA levels in the group of patients without UVB irradiation. Furthermore, the level of MBD2 expression was negatively correlated with DNA methylation in patients with SLE without UVB irradiation and positively correlated with SLEDAI. Due to the demethylation effect of MBD2, the increased expression of MBD2 mRNA may be associated with DNA hypomethylation in patients with SLE. This is compatible with the study by Balada *et al* (43).

DNA methylation decreased following irradiation with various dosages of UVB. Moreover, DNA methylation levels decreased significantly in patients with active SLE following a low dose of UVB (50 mJ/cm^2^) radiation, but decreased significantly only following a high dose of UVB (100 mJ/cm^2^) radiation in the non-active SLE patients and controls. The mRNA levels of DNMT1 decreased significantly after the high dose of UVB (100 mJ/cm^2^) radiation in the patients with active SLE, while no significant differences were observed in the non-active SLE patients and controls following the two radiation dosages. No significant differences were observed in the levels of MBD2 mRNA following the different dosages of UVB irradiation in the patients and controls. Patients with active SLE appear to have a higher sensitivity to UVB exposure that results in significant decreases in DNA methylation levels, suggesting that UVB exposure may have a role in the pathogenesis of SLE by decreasing DNA methylation, which may be caused by the decreased expression of DNMT1. This is consistent with the clinical phenomenon whereby active patients are more sensitive to sunlight.

In the present study, neither DNA hypomethylation nor the mRNA levels of DNMT1 and MBD2 were accounted for by the type of medication the patients were taking, with or without UVB irradiation. Certain studies have reported that the drugs commonly used to treat lupus do not inhibit DNA methylation (44,45); changes in the methylation status of SLE patients are unlikely to be the result of corticosteroid treatment (44,46) and the mRNA expression levels of DNMT1, DNMT3a, DNMT3b, MBD2 and MBD4 are not affected (43,44). Similar results in which there were no differences between non-treated patients and patients who had received medication (such as prednisone and cyclophosphamide) irrespective of UVB irradiation were observed in the present study.

It has been reported that SLE patients in remission have normal DNA methylation levels and reduced amounts of 5-methylcytosine are observed in the T-cell DNA of active patients, but not non-active patients (44). A negative correlation was also observed between methylation and SLEDAI iin the current study. No correlation were observed between the laboratory parameters tested and DNA methylation or the mRNA levels of DNMT1 and MBD2. In one study (46), it was noted that T cells from patients with lupus exhibit diminished levels of DNMT activity. Another study (47) suggested that the decrease in DNMT enzyme activity is due to a decrease in the level of the DNMT mRNA. It has been observed that the mRNA levels of the MBDs (MBD2 and MeCP2) involved in the DNA methylation process are significantly higher in lupus patients (48). We suggest that the laboratory parameters may reflect the disease activity but do not completely parallel the fundamental defects, such as the epigenetics of the disease, and the global DNA methylation may depend on multiple factors, such as the DNMT mRNA levels, DNMT activity, MBD mRNA levels and the transcript levels of other enzymes involved on the DNA methylation process. It is possible that a complicated regulatory mechanism for these enzymes may be involved in human T cells.

In conclusion, epigenetic changes appears to be important in the regulation of SLE. Global DNA hypomethylation has an important role in the pathogenesis of SLE. Lower expression levels of DNMT1 mRNA and higher expression levels of MBD2 mRNA may be involved in the pathogenesis of SLE, but are not the only regulation factors for global DNA methylation in SLE. DNA methylation and DNMT1 mRNA expression levels decrease in patients with active SLE following UVB exposure, indicating that UVB may have a role in the pathogenesis of SLE. The mechanism of the effect of UVB on DNA methylation in SLE patients is complicated and should be studied further. This may provide important evidence for assisting in the development of new treatments for SLE through sun protection, as well as providing a deeper understanding of the etiology of this disease.

## Figures and Tables

**Table I:** Level of DNA methylation following various dosages of UVB radiation in SLE patients and controls.

Group	Cases	0 mJ/cm^2^	50 mJ/cm^2^	100 mJ/cm^2^
Active SLE	16	8.469±1.458	7.061±1.326^a^	6.148±1.324^a^
Non-active SLE	19	11.090±1.418	10.186±1.385	8.276±1.411^a^
Controls	21	11.479±1.291	10.869±1.326	9.883±1.408^a^

Values shown represent the mean ± standard deviation.

a Statistically significant difference in DNA methylation level compared with 0 mJ/cm^2^ (P<0.01). SLE, systemic lupus erythematosus.

**Table II:** Level of DNMT1 mRNA following various dosages of UVB radiation in SLE patients and controls.

Group	Cases	0 mJ/cm^2^	50 mJ/cm^2^	100 mJ/cm^2^
Active SLE	16	0.449±0.160	0.402±0.137	0.364±0.127^a^
Non-active SLE	19	0.514±0.154	0.494±0.144	0.488±0.140
Controls	21	0.762±0.194	0.734±0.191	0.701±0.161

Values represent the mean ± standard deviation.

a Statistically significant difference in DNMT1 mRNA level compared with 0 mJ/cm^2^ (P<0.05). DNMT1, DNA methyltransferase 1; SLE, systemic lupus erythematosus.

**Table III:** Level of MBD2 mRNA following various dosages of UVB radiation in SLE patients and controls.

Group	Cases	0 mJ/cm^2^	50 mJ/cm^2^	100 mJ/cm^2^
Active-SLE	16	1.360±0.310	1.334±0.303	1.386±0.312
Non-active SLE	19	1.074±0.274	1.104±0.280	1.124±0.296
Controls	21	0.970±0.269	0.966±0.283	0.991±0.284

Values represent the mean ± standard deviation. No statistically significant difference in MBD2 mRNA level compared with 0 mJ/cm^2^ (P>0.05). MBD2, methyl CpG binding domain protein 2; SLE, systemic lupus erythematosus.
